# Preoperative bowel preparation promotes intestinal functional recovery after esophagectomy

**DOI:** 10.4314/ahs.v23i3.62

**Published:** 2023-09

**Authors:** Junliang Ma, Shaolin Chen, Xiaoying Ren, Hao Han, Ming Gong, Yongxiang Song, Lijuan Liu

**Affiliations:** 1 Department of thoracic surgery, Affiliated Hospital of Zunyi Medical University, Zunyi, Guizhou 563003, P.R. China; 2 Nursing Department, Affiliated Hospital of Zunyi Medical University, Zunyi, Guizhou 563003, P.R. China; 3 Department of thoracic surgery, The Second Affiliated Hospital of Xi'an Jiaotong University, Xian, Shaanxi 710004, P.R. China

**Keywords:** intestinal dysfunction, esophagectomy, retrospective cohort study

## Abstract

**Background:**

Patients are prone to intestinal dysfunction after esophagectomy. The value of preoperative bowel preparation before esophagectomy is controversial. There is a lack of evidence as to whether preoperative bowel preparation can help patients improve bowel function and shorten the recovery time of bowel function.

**Objectives:**

The objectives of this study were to explore whether preoperative bowel preparation can promote the recovery of intestinal function after esophagectomy.

**Methods:**

We analysed 139 patients who underwent elective radical esophagectomy in the Department of Thoracic Surgery at the Second Affiliated Hospital of Xi'an Jiaotong University from May 2016 to December 2018. The enrolled patients were divided into the study group (bowel preparation group) and the control group (no bowel preparation group) of 71 cases and 68 cases. Patients in the study group were given dissolved polyethylene glycol electrolyte powder and a cleansing enema the day before surgery. Patients in the control group were neither given polyethylene glycol electrolyte powder nor cleansing enemas before surgery. The postoperative recovery of the two groups were compared.

**Results:**

Postoperative bed rest time, bowel function recovery time and the time of first flatus and defecation after surgery were significantly shorter in patients with bowel preparation than in those without bowel preparation, and the differences were statistically significant. (P=0.038, P<0.001, P<0.001, P<0.001; respectively).

**Conclusions:**

Preoperative bowel preparation can promote the recovery of patients with esophageal cancer, especially the recovery of bowel function, which can reduce the pain caused by abdominal distension and improve the quality of life of patients.

## Introduction

Esophageal cancer (EC) is a serious threat to human health with an overall 5-year survival rate of less than 20%^1^, making it the 9th and 6th most common cause of morbidity and mortality worldwide, respectively^2^.

In China, the number of new cases of EC accounts for more than half of the total new cases in the world. The main treatment for EC remains surgical resection^3^. Although modern advances in anesthesia and surgical techniques have reduced the risk of esophagectomy, surgically complex esophagectomy is still characterized by high trauma and complications^4^-^7^.

According to the national clinical database, the incidence of postoperative complications after esophagectomy is about 43% and the postoperative mortality rate is about 3%^8^. Moreover, after esophagectomy, patients are prone to symptoms such as pain, fatigue, anorexia and digestive system diseases^9^,^10^, so intestinal dysfunction is easy to occur after esophagectomy. Methods to reduce surgical risks and postoperative complications are still controversial^11^,^12^. Preoperative bowel preparation is not a necessary procedure in esophagectomy, and whether to perform it remains controversial. The Enhanced Recovery After Surgery (ERAS) concept suggests that oral laxatives and enemas can cause or exacerbate preoperative dehydration and electrolyte balance disturbances, so enema bowel preparation is not recommended^13^,^14^. However, bowel preparation removes food debris from the intestine, stimulates peristalsis, softens bowel movements, eliminates intestinal gas, reduces bloating, and cleanses the intestine^15^,^16^. It has the potential to reduce intestinal complications after esophagectomy.

Whether bowel preparation can help patients improve intestinal function and shorten the recovery time of intestinal function is still lacking. The purpose of this study was to evaluate preoperative cleansing enemas as a potential strategy to promote recovery of bowel function after esophagectomy.

## Materials and Methods

### Ethics

The study was conducted in accordance with the Declaration of Helsinki and approved by the research ethics committee of the Second Affiliated Hospital of Xi'an Jiaotong University. This study was conducted in accordance with the principles of the Declaration of Helsinki.

### Study populations

A retrospective cohort study was conducted on EC patients undergoing elective esophagectomy. A total of 139 patients were enrolled in this study between May 2016 and December 2018 at the Department of Thoracic Surgery, Second Affiliated Hospital of Xi'an Jiaotong University. Patients who met the diagnostic criteria of esophageal cancer and were diagnosed with squamous esophageal cancer of the thoracic segment by gastroscopy and histological biopsy pathology before surgery; 2. Patients aged 40-75 years; 3. Patients with clear indications for surgery and no contraindications to surgery by imaging, hematological examination and cardiopulmonary function assessment before surgery; 4. Patients with preoperative clinical stage of cT1-3N0-1M0. Exclusion criteria: 1. Patients with intraoperative use of the colon or small intestine instead of the esophagus for esophageal cancer; 2. Patients with gastrointestinal obstruction, intestinal or gastric retention, or a history of abdominal surgery. The enrolled patients were divided into study group (bowel preparation group) and control group (no bowel preparation group) with 71 and 68 cases.

### Bowel cleansing methods

The two groups of patients were prohibited from eating vegetables and fruits 2 days before surgery. Patients in the study group were given dissolved polyethylene glycol electrolyte powder and cleansing enema one day before surgery. The procedure was as follows: after lunch the day before surgery, polyethylene glycol electrolyte powder was dissolved in 1,000 mL of water and taken twice in half an hour.

A cleansing enema was performed at 8 pm using a disposable silicone anal tube of 4.67 mm. The enema solution consisted of 20 ml of a 10% soft soap solution and 1,000 mL of warm boiled water. Patients fasted after 10 p.m. the day before the procedure. Patients in the control group did not take either polyethylene glycol electrolyte powder or cleansing enemas before the procedure.

In EC surgery, transnasal duodenal feeding tubes are routinely placed in all surgical patients. On the 1st day after the operation, glucose and sodium chloride injection was injected through the feeding tube, and the enteral nutrition solution was injected through the feeding tube on the 3rd to 4th postoperative day. During the study period, in order to prevent the occurrence of anastomotic leak, patients with esophageal cancer were given esophagography 10 days after surgery. When the angiography results showed that there was no anastomotic leak, the patient had good gastrointestinal function and no obvious signs of local or systemic infection, oral feeding was administered.

### Data Collection

Patient demographic and clinical data collected included gender, year of birth, smoking, alcohol consumption, body mass index (BMI), tumor size, tumor location, type of pathology, clinical stage and surgical approach. Postoperative variables were collected, including time to start walking, time to recovery of bowel movements, time to anal discharge, time to first bowel movement, time to oral food intake, bloating, constipation, bowel obstruction, anastomotic leak, celiac disease, lung infection, chest tightness, shortness of breath and length of hospital stay. The incidence of nausea, vomiting, abdominal pain, bloating and other adverse reactions after drug administration were evaluated.

### Statistical analysis

SPSS 19.0 statistical software (IBM Corporation, Armonk, NY, USA) was used for statistical analysis of the data. Continuous variables were expressed as mean ± standard deviation, and t-test was used for comparison of 2 independent samples. Enumeration data were described by the number of cases (percentage) and tested by Pearson's chi-square or Fisher's exact test. P value < 0.05 was considered statistically significant.

## Results

From May 2016 to December 2018, a total of 139 patients were enrolled in the study and underwent esophagectomy in the Second Affiliated Hospital of Xi'an Jiaotong University. The surgical approach consisted mainly of Sweet and McKeown. Among 120 cases of McKeown, 57 underwent minimally invasive esophagectomy (MIE). Radical resection (R0) was achieved in all patients. Clinicopathological characteristics, including age, gender, smoking, alcohol consumption, BMI, tumor size, tumor location, histology, clinical stage and surgical approach were comparable between the two groups.

Patient characteristics and surgical features are shown in Table I. A total of 139 patients were included in the study, and the ages of these patients ranged from 31 to 81 years. The mean age of all patients was 64.0 years (SD=8.94). There were 115 men and 24 women in the study population, 71 (51.1%) of whom received bowel preparation. Most of the EC patients had a habit of smoking and/or drinking. The esophageal tumors were mostly located in the lower thoracic segment and were histologically predominantly esophageal squamous cells. The main clinical stage of the patients was stage II. As shown in Table 1, these clinicopathological features were not significantly different between the two groups (P > 0.05).

**Table 1:** Baseline and clinical Characteristics of the study population. Sweet: left thoracic, one incision. McKeown: Three Incision Esophagectomy (Laparotomy, Right Thoracotomy with Cervical Anastomosis). MIE: minimally invasive esophagectomy

Characteristics	N	Bowel preparation		x^2^/t value	P value

		Yes	No		
Gender				0.014	0.907
Male	115	59	56		
Female	24	12	12		
Age		63.85±8.76	64.09±9.18	0.160	0.873
Smoking				0.008	0.929
Yes		57	55		
No		14	13		
Drinking				0.115	0.831
Yes		58	54		
No		13	14		
BMI		21.42±1.24	21.10±1.63	-1.311	0.190
Tumor size		5.65±2.13	5.79±2.41	0.360	0.719
Tumor location				4.555	0.103
Upper		1	2		
Middle		27	37		
Lower		43	29		
Histology				1.064	0.587
Neuroendocrine carcinoma		0	1		
Adenocarcinoma		10	9		
Squamous cell carcinoma		61	58		
Clinical stage				2.349	0.503
?		7	4		
?		49	46		
?		14	18		
?		1	0		
Surgical method				1.245	0.537
Sweet		10	9		
Mckeown (MIE)		32	25		
Mckeown		29	34		

The postoperative outcomes of EC patients are presented in Table 2. No patient died within 30 days postoperatively. The postoperative bed rest time, bowel function recovery time, anal venting time, and first bowel movement time were significantly shorter in patients with bowel preparation than in patients without bowel preparation, and the differences were statistically significant. (P = 0.038, P<0.001, P<0.001, and P<0.001; respectively). The incidence of abdominal distension and constipation was lower in the group with bowel preparation than in the group without bowel preparation (P < 0.001; P = 0.021).

**Table 2:** Postoperative Outcomes

Variables	Bowel preparation		x^2^/t value	P value

	Yes	No		
Bedtime	2.056±0.984	2.514±1.550	2.091	0.038
Recovery time of intestinal peristalsis	2.254±1.052	3.838±1.512	7.198	< 0.001
Anal exhaust time	2.803±1.305	4.427±1.396	7.705	< 0.001
First defecation time	4.239±1.871	5.368±1.53	3.895	< 0.001
Oral feeding time	12.282±12.856	13.824±10.856	0.765	0.446
Abdominal distention			18.772	< 0.001
Yes	18(25.4%)	42(61.8%)		
No	53	26		
Constipation			5.892	0.021
Yes	9(12.7%)	20(29.4%)		
No	62	48		
Intestinal obstruction			2.119	0.238
Yes	0 (0)	2(2.9%)		
No	71	66		
Anastomotic leakage			1.916	0.201
Yes	3(4.2%)	7(10.3%)		
No	68	61		
Chylothorax			1.052	0.489
Yes	0 (0)	1(1.5%)		
No	71	67		
Pulmonary infection			3.575	0.073
Yes	3(4.2%)	9(13.2%)		
No	68	59		
Shortness of breath			3.349	0.085
Yes	6(8.5%)	13(33.8%)		
No	65	55		
Postoperative hospital stay (mean ± sd), days	16.887±13.568	22.632±35.842	1.239	0.210
30-day mortality	0	0		

The incidence of anastomotic leakage, chylothorax, pulmonary infection and shortness of breath were lower in the group with bowel preparation than in the group without bowel preparation, but there was no significant difference between the two groups (P > 0.05). The postoperative hospital stay was shorter in patients with bowel preparation than in patients without bowel preparation however, the difference was not statistically significant (P > 0.05).

We further compared the duration of abdominal distension, constipation, anastomotic fistula, pulmonary infection, chest tightness and shortness of breath in both groups. As we can see in Table 3, the duration of abdominal distension, constipation, anastomotic leakage, pulmonary infection and chest shortness of breath were shorter in patients with bowel preparation than in patients without bowel preparation. However, only the comparison of the duration of pulmonary infection was statistically significant.

**Table 3:** The duration of abdominal distension, constipation, anastomotic fistula, pulmonary infection and shortness of chest tightness in the two groups

Variables	Bowel preparation		t value	P value

	Yes(n)	No(n)		
Duration of abdominal distension (days)	2.053±1.471(18)	2.833±1.962(42)	1.722	0.092
Duration of constipation (days)	1.889±1.167(9)	2.750±2.291(20)	1.339	0.192
Duration of anastomotic fistula (days)	29.330±9.018(3)	32.71±15.03(7)	0.439	0.675
Duration of pulmonary infection (days)	15.000±1.000(3)	17.857±2.854(9)	2.335	0.038
Duration of shortness of breath (hours)	24.143±18.115(6)	46.154±35.949(13)	1.820	0.085

## Discussion

After esophagectomy, patients have gastric tubes, nutrition tubes, oxygen tubes, deep venous catheters, closed chest drains, and urinary catheters, which restrict patients' activities, prolong their bed rest, delay the recovery of bowel function, and may bring a series of problems.

The ideal bowel preparation is to achieve intestinal cleaning, at the same time, it has little effect on physiological function, short duration, less pain, and is easy to be considered by patients. Polyethylene glycol electrolyte powder is a widely used intestinal cleaner. Its active ingredient is polyethylene glycol 4000, which combines with water molecules to form a more stable hydrogen bond^17^-^19^. When the drug reaches the intestine, polyethylene glycol electrolyte powder prevents water in the intestinal contents from being excessively absorbed by the colon, thereby lubricating the intestine, softening stool, increasing the volume of intestinal contents, and promoting normal physiological movement of the colon. It is recognized as a first-line cleaning agent with high efficiency and safety^20^,^21^.

As a volumetric laxative, it cleanses the intestine by emptying the digestive juices. This method does not interfere with intestinal absorption and secretion, nor does it cause water and electrolyte disturbances. Patients were given a single enema after oral administration of polyethylene glycol electrolyte powder, which increased the degree of bowel cleansing. Patients in the bowel preparation group were able to successfully complete the oral administration of polyethylene glycol electrolyte powder and enemas with good compliance and no serious adverse effects.

Our research results show that the postoperative bed rest time, bowel function recovery time, anal exhaust time, and first defecation time for patients with EC undergoing bowel preparation were significantly shorter than those without bowel preparation. The incidence of abdominal distension and constipation in the bowel preparation group were lower than that in the non-intestinal preparation group. These results suggest that preoperative bowel preparation can promote the recovery of patients with EC, especially the recovery of intestinal function and can reduce the pain caused by abdominal distention and improve the quality of life of patients.

In accordance with previous studies, postoperative pneumonia appeared to be the most common infectious complication in patients following esophagectomy7,22. Although cleaning the intestines does not reduce the incidence of pulmonary infection, it can reduce the duration of pulmonary infection. The reason may be that the patients' bed time is shortened and the intestinal bacterial migration is reduced.

Taken together, cleaning enema is a non-invasive operation with good safety, easy to accept by patients. This group of research data shows that cleaning enema before radical operation of esophageal cancer can promote patients to recovery of intestinal function, which can be used as routine before radical operation of esophageal cancer.

## Data Availability

The datasets used during the current study are available from the corresponding author on reasonable request.

## Fundings

This work is supported by grants from the Science and Technology Foundation of Guizhou Province (Qian Ke He J-ZK [2021] General 465), Doctor start-up Fund of Zunyi Medical University (Yuan Zi [2020] No. 08), Health Commission of Guizhou Province (gzwjkj2020-1-108).
